# Application of a Closed-Form Model in Analyzing the Fracture of Quasi-Brittle Materials

**DOI:** 10.3390/ma17020282

**Published:** 2024-01-05

**Authors:** Xiangyu Han, Peng Li, Jianguo Liu

**Affiliations:** 1 School of Civil Engineering and Architecture, Southwest University of Science and Technology, Mianyang 621010, China; 2 China Railway 17th Bureau Group Co., Ltd., Taiyuan 030006, China; 15882103924@163.com

**Keywords:** quasi-brittle materials, boundary effect, fracture analysis, microstructure

## Abstract

Fracture failure in quasi-brittle materials poses a persistent challenge in materials science and engineering. This study presents a thorough investigation of the Boundary Effect Model (BEM), offering a nuanced understanding of the size effect on fracture properties. The conceptual framework, evolutionary process, and applicability scope of BEM are elucidated, highlighting its accuracy and reliability in calculating fracture properties across various quasi-brittle materials. Through the integration of BEM with diverse fracture tests—such as three-point bending, four-point bending, and wedge-splitting—a linear correlation between maximum failure loads and material fracture properties is established. Notably, the study demonstrates that fracture properties, determined by BEM, can be regarded as consistent material constants across specimens of varying sizes, initial notch lengths, geometries, and microstructures. Validation of the BEM’s reliability encompasses the analysis of 140 fracture test results involving concrete, hard rocks, and bamboo scrimber. The synergy of non-linear and linear BEM analyses emerges as a robust approach for accurately predicting the fracture behavior of quasi-brittle materials. This comprehensive exploration sheds light on the potential of the Boundary Effect Model as a valuable tool for predicting and understanding fracture mechanics in diverse materials and scenarios. This research serves as an effective approach to accurately evaluating the fracture properties of quasi-brittle materials, which is of great practical significance for material design, engineering construction, and various industrial applications.

## 1. Introduction

Quasi-brittle materials, such as concrete, hard rocks, brickwork, stiff clay, etc., not only exhibit brittle behavior but also possess some degree of toughness or ductility. These materials often have low fracture toughness compared to ductile materials but still have some ability to deform and absorb energy before fracturing [1,2,3]. This is due to the presence of unique microstructures such as aggregates, pores, cracks, and voids, which can act as sites for energy dissipation and plastic deformation [4,5].

When loaded, quasi-brittle materials tend to fail by cracking rather than yielding or deforming plastically. Compared with brittle or ductile materials, the fracturing behavior of quasi-brittle materials is more complex and is always influenced by various factors. Firstly, the mode and magnitude of loading will affect the fracture behavior of quasi-brittle materials. Tension, compression, and shear loading can cause different fracture modes and failure mechanisms [6]. Higher loading rates will cause more brittle fractures, while slower loading rates will induce ductile behavior. Secondly, the fracture of quasi-brittle materials has been proven to be directly related to the specimen geometry and size; the presence of pre-existing defects will also weaken the fracture toughness of specimens [7]. Thirdly, environmental conditions, such as temperature, humidity, and chemical exposure, will change the fracture performance of quasi-brittle materials [8,9]. When exposed to high temperatures or aggressive chemicals, the quasi-brittle materials will be degraded and more likely to fracture. Last, the microstructures are another important influencing factor, such as the aggregate size, microcracks, pores, and voids [10,11].

Then, several methods were proposed to study the fracture of quasi-brittle materials, such as experimental methods, theoretical methods, and numerical simulation analysis. Corr et al. [12] applied digital image correlation (DIC) to observe the development of the fracture process zone in full-graded dam concrete. Han et al. [13] used X-ray microscopy to reveal the cracking features underneath the fracture surface. On the basis of these experimental results, different theoretical models were established. Hillerborg et al. [14] considered the stress-transferring crack as a fictitious crack and proposed the fictitious crack model to depict the stress distribution around the crack tip. Bazant [15,16] attributed the size effect to the variation of length and the area of the crack band and established a size effect law to illustrate the fracture behavior of concrete and rock. Hu [17,18] found that the size effect of fracture properties of quasi-brittle materials is mainly caused by the variation of specimen boundary conditions and proposed the Boundary Effect Model to predict the fracture of quasi-brittle materials. Xu et al. [19,20] regarded the quasi-brittle fracture as composed of two states and utilized the initial cracking toughness and the unstable fracture toughness to distinguish the fracture process of quasi-brittle materials. Furthermore, numerical simulation analysis is also adopted to study the fracture of quasi-brittle materials, such as the Particle Flow Code, Abaqus, Three-Dimensional Distinct Element Code, etc., and obtain some meaningful results [21,22,23]. 

It could be found that fracture is one of the main failure modes of quasi-brittle materials; the precise description and prediction of fracture are critical during the application of quasi-brittle materials. The current research seeks to offer solutions to this pressing issue by providing comprehensive methodologies for analyzing the fracture of quasi-brittle materials. The framework of the whole paper is arranged as follows: Section 2 mainly describes the proposal of the BEM concept; Section 3 mainly illustrates the closed-form solutions for different testing methods; Section 4 presents the application of BEM in various quasi-brittle materials; Section 5 gives the discussion and Section 6 provides the conclusions.

## 2. The Concept of the Boundary Effect Model

**Figure 1:**
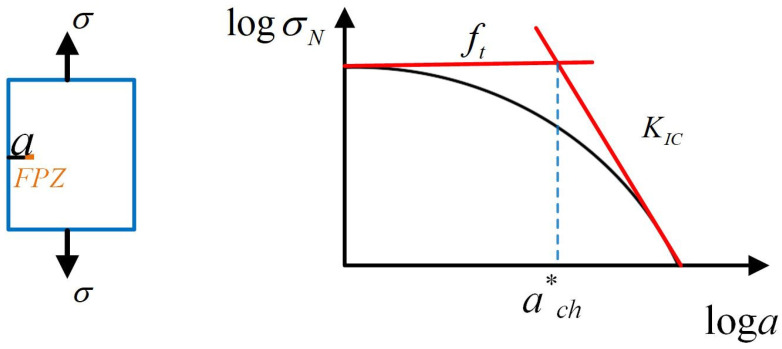
The asymptotic quasi-brittle fracture curve of a large plate with a short edge crack.

It is widely acknowledged that the fracture properties of quasi-brittle materials, as estimated by the traditional linear elastic fracture theory, are subject to variation with changes in specimen size. Hu [24] thought the above-size effect was similar to the well-studied elastic plastic fracture of metals and attributed such a phenomenon to the effect of cracking close to the free surface. Then, the asymptotic analysis approach was established on the basis of the elastic/plastic fracture transition of a large plate with a small edge crack, as shown in Figure 1 [25]. Here, the asymptotic equation is determined by the yield strength *σ_Y_* criteria (a horizontal line) and fracture toughness *K_IC_* criteria (an oblique line).

For a large plate with a small edge crack, the asymptotic equation can be described as follows:

(1) $$\sigma_{N}=\frac{f_{t}}{\sqrt{1+\frac{a}{a_{ch}^{*}}}}$$

where *σ_N_* is the nominal stress, *f_t_* is tensile strength, and *a* is the initial crack length. It can be clearly observed that, when $a/a_{ch}^{*}\ll 1$, the failure of a plate is mainly controlled by the strength criterion, when $a/a_{ch}^{*}\gg 1$, the failure is mainly controlled by the fracture toughness criterion. The characteristic crack length $a_{ch}^{*}$ is defined by the intersection of the two criteria mentioned above, which is a material constant determined according to Equation (2) [24]. Where *Y* is the geometry factor, which is always set at 1.12 for infinite plates.

(2) $$a_{ch}^{*}=\frac{1}{\pi Y^{2}}{\left(\frac{K_{IC}}{f_{t}}\right)}^{2}=0.25{\left(\frac{K_{IC}}{f_{t}}\right)}^{2}$$

However, finite-sized specimens are more commonly encountered than infinite plates in the real scenario. For the fracture issues in finite-sized specimens, the initial crack length in Equation (1) should be replaced by the equivalent crack length *a_e_*, as shown in Equations (3) and (4) [18].

(3) $$a_{e}={\left[\frac{{\left(1-\alpha \right)}^{2}\times Y(\alpha )}{1.12}\right]}^{2}a_{0}\;(3-p-b\;\mathrm{and}\;4-p-b\;\mathrm{specimens})$$

(4) $$a_{e}={\left[\frac{{\left(1-\alpha \right)}^{2}\times Y(\alpha )}{0.56(2+\alpha )}\right]}^{2}a_{0}(\mathrm{WS}\;\mathrm{specimens})$$

where *a*_0_ is the initial crack length of specimens, *α* is the ratio of *a*_0_ to specimen height *W*, and *Y*(*α*) is the geometry factor determined by *α* and specimen geometry. In that way, the effect of the specimen boundary is carefully considered. The nominal stress (*σ_n_*) of finite-sized specimens can be illustrated in Equation (5).

(5) $$\sigma_{n}=\frac{f_{t}}{\sqrt{1+\frac{a_{e}}{a_{ch}^{*}}}}$$

Actually, the effect of the specimen boundary on determining the fracture toughness of materials has been reflected in several standards. In American Society for Testing and Materials (ASTM) E399, the specimens for testing fracture toughness are required to meet the conditions in Equation (6) [26].

(6) $$\begin{array}{l} B\\ a_{0}\\ W-a_{0} \end{array}\}\geq 2.5{\left(\frac{K_{IC}}{f_{t}}\right)}^{2}$$

By referring to Equations (2) and (5), the distance between crack tip and specimen boundary is considerable, the fracture toughness criterion can be applied, and the tested fracture toughness is reliable. In International Union of Laboratories and Experts in Construction Materials, Systems, and Structures (RILEM) TC-50FMC, the initial crack length, specimen size, and specimen thickness are all specified [27]. With the increase in aggregate size, a notable increase is required for the specimen sizes. In this way, the fracture properties of concrete materials can be obtained as constants.

## 3. The BEM in Different Fracture Tests

There are several methods for testing the fracture behavior of quasi-brittle materials, such as the three-point bending test, the four-point bending test, the wedge-splitting test, etc. In these fracture tests, the initial notch is always introduced into the specimens during specimen preparation. Then, the fracture will occur along the desired path, thereby ensuring the integrity and reliability of the results. Moreover, the length of the initial notch should consider the effect of material microstructure and specimen size. 

Before analyzing the stress distribution around the notch tip and introducing BEM, there are a few assumptions that need to be made: (1) During the fracture process of the specimen, the unfractured part remains horizontal under the action of the load, and the strain distribution along the height of the unfractured region is linear. (2) The compressive and tensile stresses in the unfractured region are linearly distributed along the height of the beam. (3) The tensile elastic modulus of quasi-brittle materials is equal to the compressive elastic modulus. (4) The crack bridging stress is constant within the partially developed fracture process zone (fictitious crack) [28]. 

**Figure 2:**
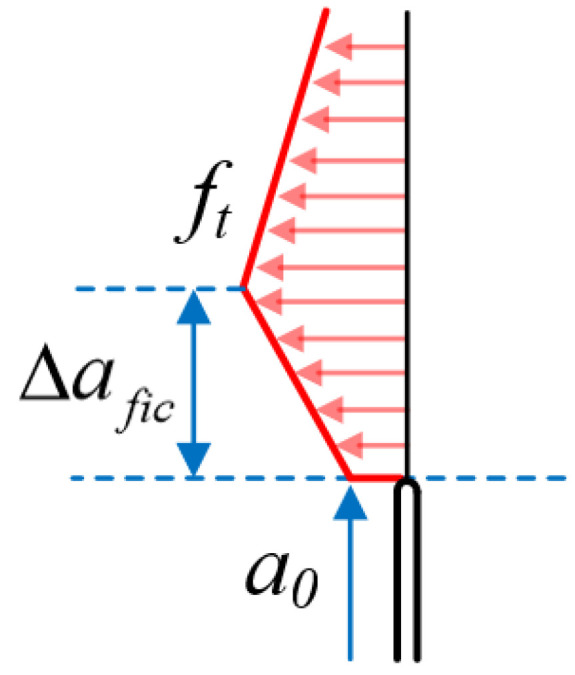
The stress distribution around the notch tip.

The first three assumptions are commonly used in other fracture models, so this section mainly focuses on the fourth assumption. According to Hillerborg’s fictitious crack model, the stress values gradually decrease from tensile strength to zero along the fictitious crack path, and such a type of fracture process zone is called a fully developed fracture process zone. However, in laboratory tests, the pre-existing crack tips are always large and blunt compared to the real tip of a propagating crack. As shown in Figure 2, the stress at the crack tip is a non-zero value, and the fracture process zone formed at the pre-existing crack tip is not fully developed [29]. The distance from the pre-existing crack tip to the point where the stress equals the material’s tensile strength (the unfractured region) is defined as the fictitious crack length (∆*fic*). 

Considering the physical meaning of the fictitious crack, it can be seen that the fictitious crack length is a physical quantity related to the aggregate particle size and is less than the length of the fully developed fracture process zone [30]. The fictitious crack length can be described as Equation (7):

(7) $$∆a_{fic}=\beta G$$

According to RILEM TC-50FMC, for laboratory-conducted fracture tests, the quasi-brittle specimen size/aggregate size (W/G) is approximately equal to 10, and the unnotched ligament length/aggregate size ((W − *a*_0_)/G) is only 5. Moreover, fictitious cracks only occur in the tensile zone of the specimen’s ligament. Therefore, the parameter (β) in Equation (7) could be set between 1 and 2. Since the size of the fictitious crack length and the crack opening displacement of quasi-brittle specimens near failure are both small, the stress value in such an incompletely developed fracture process zone can be regarded as constant. 

### 3.1. Three-Point Bending (3-p-b) Tests

**Figure 3:**
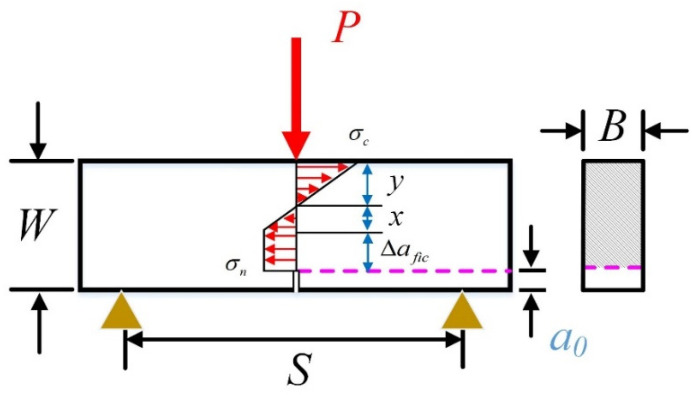
The stress distribution of three-point bending specimens.

As shown in Figure 3, the stress distribution along the un-notched ligament of three-point bending specimens is illustrated on the basis of the above assumptions. It can be found that the ligament is composed of compressive area (*y*) and tensile area (*x* + ∆*fic*). It should be noted that the stress in the fictitious crack area (∆*fic*) is assumed to be constant, as depicted above. After that, the relation between maximum tensile stress ($\sigma_{n}$) and peak load (*P_max_*) can be established with four assumptions: the equilibrium theories of forces and moments [29]. The detailed derivation process is presented in Equations (8)–(14).

(8) $$\frac{\sigma_{c}}{\sigma_{n}}=\frac{y}{x}$$

(9) $$x+y=W-a_{0}-∆a_{fic}$$

(10) $$\frac{1}{2}\sigma_{c}\cdot y\cdot B=\frac{1}{2}\sigma_{n}\cdot x\cdot B+\sigma_{n}\cdot ∆a_{fic}\cdot B$$

(11) $$\frac{1}{2}P_{\max}\cdot \frac{1}{2}S=\frac{1}{2}\sigma_{c}\cdot y\cdot \frac{2}{3}y\cdot B+\frac{1}{2}\sigma_{n}\cdot x\cdot \frac{2}{3}x\cdot B+\sigma_{n}\cdot ∆a_{fic}\cdot (\frac{∆a_{fic}}{2}+x)\cdot B$$

(12) $$\sigma_{n}=\frac{1.5\cdot \frac{S}{B}\cdot P_{\max}}{\left(W-a_{0})(W-a_{0}+2∆a_{fic}\right)}$$

Then, incorporating Equation (12) into Equation (5), the relation between tensile strength and *P_max_* of the 3-p-b tests is constructed as follows:

(13) $$P_{\max}=f_{t}\cdot \frac{\left(W-a_{0})(W-a_{0}+2∆a_{fic}\right)}{1.5\cdot \frac{S}{B}\cdot \sqrt{1+\frac{a_{e}}{a_{\infty }^{*}}}}$$

For the specimens with different *S/W* ratios, their geometry factors *Y*(*α*) can be obtained with Equations (14)–(16) [31].

(14) $$Y_{2.5}(\alpha )=\frac{1-2.5\alpha +4.49\alpha^{2}-3.98\alpha^{3}+1.33\alpha^{4}}{{\left(1-\alpha \right)}^{3/2}}\;(S/W=2.5)$$

(15) $$Y_{4.0}(\alpha )=\frac{1.99-\alpha (1-\alpha )(2.15-3.93\alpha +2.7\alpha^{2})}{\sqrt{\pi }(1+2\alpha ){\left(1-\alpha \right)}^{3/2}}\;(S/W=4.0)$$

(16) $$Y_{8.0}(\alpha )=1.107-2.12\alpha +7.71\alpha^{2}-13.55\alpha^{3}+14.25\alpha^{4}\;(S/W=8.0)$$

### 3.2. Four-Point Bending (4-p-b) Tests

**Figure 4:**
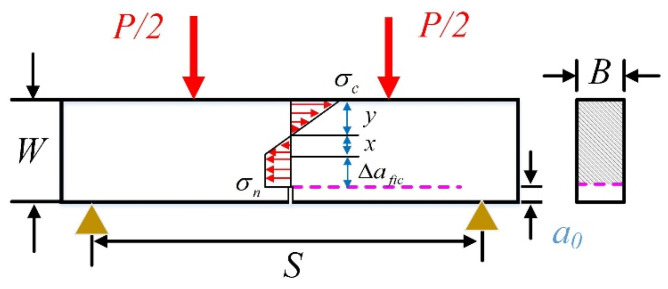
The stress distribution of four-point bending specimens.

The 4-p-b tests are also commonly used fracture test methods that have been applied to different quasi-brittle materials, such as concrete, hard rock, etc. [32,33]. On the premise of the above four assumptions, the stress distribution of test specimens is depicted in Figure 4. It can be found that the stress distribution characteristic of 4-p-b specimens is similar to that of 3-p-b specimens in Section 3.1. Compared to the 3-p-b tests, the 4-p-b test is advantageous in providing a pure bending condition for specimens, which is ideal for testing the fracture behavior of quasi-brittle materials under tensile loads. Hence, combining the stress distribution of 4-p-b specimens with BEM, the relation between tensile strength and peak loads is shown in Equation (17).

(17) $$P_{\max}=f_{t}\cdot \frac{\left(W-a_{0})(W-a_{0}+2∆a_{fic}\right)}{0.75\cdot \frac{S}{B}\cdot \sqrt{1+\frac{a_{e}}{3\cdot G}}}$$

For 4-p-b specimens, the geometry factor *Y*(*α*) can be calculated by Equation (18):

(18) $$Y(\alpha )=1.122-1.121\alpha +3.740\alpha^{2}+3.873\alpha^{3}-19.05\alpha^{4}+22.55\alpha^{5}$$

### 3.3. Wedge-Splitting (WS) Tests

**Figure 5:**
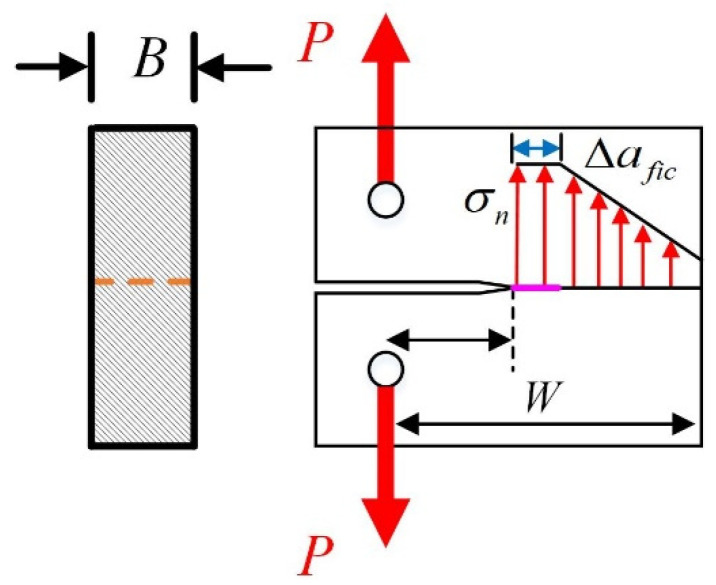
The stress distribution of wedge-splitting specimens.

The WS test is a fracture testing method that utilizes wedge-shaped tools to compel a specimen to split along a line that is perpendicular to the direction of the applied force. The biggest advantage of the WS test over the 3-p-b or 4-p-b methods is the absence of the impact of the specimen’s self-weight, especially for these specimens with heavy weight [34]. Moreover, a small vertical load can generate a larger horizontal splitting force by selecting a suitable wedge angle, thereby directly reducing the stiffness requirement for the testing machine. It should be noted that the right boundary of the specimen may suffer tensile stress or compressive stress, which depends on the specimen size and the distance between the loading point and the right boundary. As shown in Figure 5, the relationship between tensile strength and the horizontal component of *P_max_*_−*h*_ is established as follows:

(19) $$P_{\max-h}=f_{t}\cdot \frac{\left(W-a_{0})(W-a_{0}+2∆a_{fic}\right)}{\frac{2}{B}(2W+a_{0}+∆a_{fic})\cdot \sqrt{1+\frac{a_{e}}{3\cdot G}}}$$

The geometry factor of wedge-splitting specimens can be calculated as Equation (20):

(20) $$Y(\alpha )=\frac{\left(2+\alpha )(0.886+4.64\alpha -13.32\alpha^{2}+14.72\alpha^{3}-5.6\alpha^{4}\right)}{4\sqrt{\pi \alpha }{\left(1-\alpha \right)}^{3/2}}$$

## 4. Application in Various Quasi-Brittle Materials 

After the relationship between BEM and fracture test methods is established, the fracture properties of quasi-brittle materials can be easily obtained. Nevertheless, there are still some critical issues that need to be resolved.

**Figure 6:**
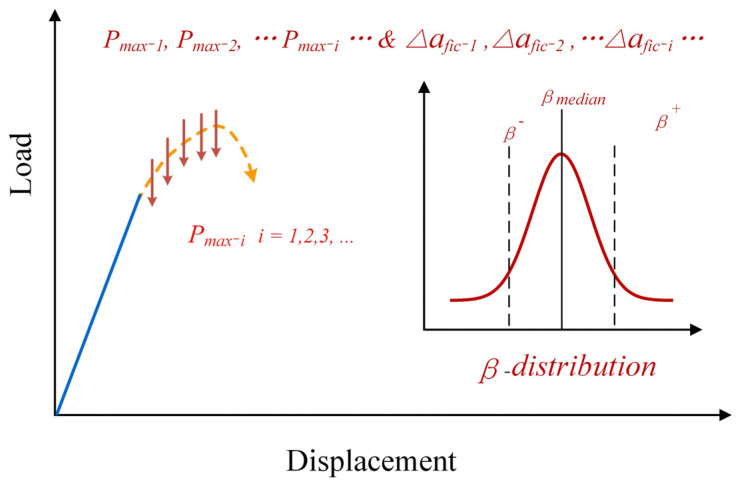
The variation of peak loads and fictitious crack lengths due to various microstructures [35].

As is known, the description of fictitious cracks is an essential part of constructing a non-linear fracture model. The length of fictitious cracks (∆*fic*) is also vital for indicating their effect on fracture test results. As illustrated in Section 3, the fictitious crack length is directly related to the microstructures of materials, and the value of *β* is approximately estimated between 1 and 2. However, the microstructures of specimens cannot be completely identical, even for one batch of specimens. Moreover, the value of *β* is always affected by the characteristics of the notch and the microstructure distribution around the notch tip. Hence, for the specimens from the same batch, the microstructures, *β* values, fictitious crack length, and maximum failure loads may be different, as shown in Figure 6. Here, the average grain (aggregate for concrete materials) *G_av_* is adopted to represent the microstructure of quasi-brittle materials; the value of *β* is recommended to be 1.5 [35,36,37], and the fictitious crack length can be calculated as Equation (21).

(21) $$∆a_{fic}=1.5G_{av}$$

It has been widely acknowledged that the scattered distribution of fracture properties is the nature of quasi-brittle materials; the obtained fracture properties will vary among specimens even for an ideal fracture model and fracture test. Such characteristics always become obstacles in determining the fracture properties of quasi-brittle materials. Actually, we do not need to determine the fracture parameter to a uniform value. On the contrary, distribution analysis can be used to calculate the distribution characteristics, and these characteristics will play vital roles in the fracture prediction of quasi-brittle materials [38,39].

As illustrated in Equation (2), the relationship between tensile strength and fracture toughness is established by the characteristic crack length, which is recommended to be three times the average grain size. Hence, the fracture toughness of quasi-brittle materials can be calculated using Equation (22).

(22) $$K_{IC}=2f_{t}\cdot \sqrt{3G_{av}}$$

In this way, the influence of specimen geometrical size on fracture test results is considered by equivalent crack length *a_e_* and geometry factor *Y*(*α*), the effect of fracture process zone is considered by microstructure *G_av_* and *β*, and the scattered distribution of fracture properties is also incorporated into the model with distribution analysis. Once the maximum failure load of one quasi-brittle specimen is tested with 3-p-b, 4-p-b, or WS tests, the tensile strength can be easily calculated with the above equations, and the fracture toughness can be determined accordingly.

### 4.1. Concrete Materials

In this section, two batches of concrete fracture test results were collected from published literature [40,41]. In these studies, the wedge-splitting method was adopted to test the fracture performance. As shown in Table 1, the wedge-splitting tests were divided into two groups (Group A and Group B). The details of the tests were summarized, and the height (W, mm), thickness (B, mm), *α*-ratio, and *P_max_* (kN) were listed. It could be found that the specimens in Group A were geometrically dissimilar (with the same α-ratios but different sizes), while the specimens in Group B were geometrically similar (with the same sizes but different *α*-ratios). It should be noted that the average aggregate size in each group is 10 and 13 mm, respectively.

**Figure 7:**
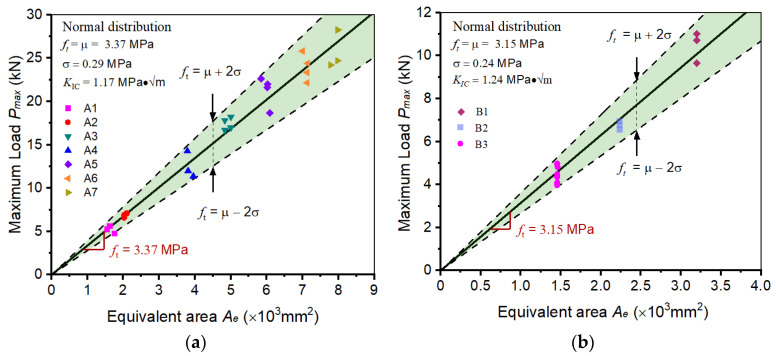
Determining the fracture properties of concrete WS specimens: (**a**) Group A and (**b**) Group B.

Then, applying these parameters to the formulas in Section 3.3, the values of tensile strength and fracture toughness can be easily determined for each specimen. Then, the normal distribution method is used to analyze the characteristics of these values. It can be observed that the distribution of tensile strength in Group A yields a mean value (*μ_a_*) of 3.37 MPa and a standard deviation (*σ_a_*) of 0.29 MPa. For the tensile strength values in Group B, the mean value (*μ_b_*) equals 3.15 MPa, and the standard deviation (*σ_b_*) equals 0.24 MPa. The standard deviation is only 8.6% and 7.6% of the mean value in Group A and Group B, which is acceptable considering the disordered microstructures of concrete and the inevitable experimental errors. The fracture toughness of concrete in Group A and Group B is calculated at 1.17 MPa·√m and 1.24 MPa·√m, respectively. By incorporating the distribution of tensile strength values into the BEM models for the WS test, the triangle-form fracture prediction method can be established, as shown in Figure 7. In Group A and Group B, most scattered data are distributed in the vicinity of the solid line, which means that the tensile strength determined by BEM and WS tests can be regarded as constants, regardless of the variation of specimen size and initial notch length.

### 4.2. Hard Rocks

In this section, the BEM is applied to determine the fracture properties of hard rocks with a 4-p-b test. Compared with the concrete-like artificial materials, the hard rocks from nature are typically formed through a geological process that involves the deposition, consolidation, and solidification of minerals and other materials over a long period of time. Consequently, uncertainty and complexity are introduced into its microstructures. Two batches of fracture test results are collected from published literature [42]. They are granite specimens (Group C) with dense structure and volcanic rock specimens (Group D) with porous structure. The details of the fracture test results are summarized in Table 2. It can be found that the specimens have the same size but different notch lengths; the span of all 4-p-b specimens is four times their height. The compressive strengths of granite and volcanic rock are 380 MPa and 48 MPa, respectively, and the porosity of volcanic rock is 0.4, which should be considered when determining the fracture properties. 

**Figure 8:**
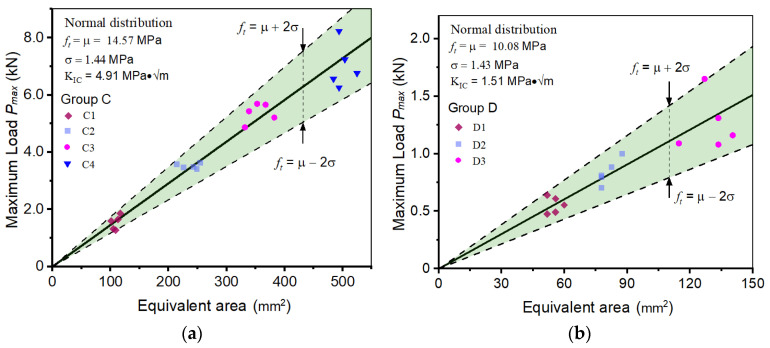
Determining the fracture properties of hard rock 4-p-b specimens: (**a**) Group C, (**b**) Group D.

Applying the established BEM to analyze the 4-p-b fracture test results in Table 2, the tensile strength of each specimen can be easily calculated, as shown in Figure 8. Then, using normal distribution analysis, analyze the distribution of the determined fracture properties. The tensile strength values in Group C and Group D are in accordance with the normal distribution of *μ_c_* = 14.57 MPa, *σ_c_* = 1.44 MPa, *μ_D_* = 10.08 MPa, and *σ_D_* = 1.43 MPa, respectively. The standard deviation is 9.9% and 14.2% of the mean values for Group C and Group D, and the tensile strength values appear to be more scattered in Group D. This may be caused by the different microstructures of different rocks; granite always has a high density and uniform crystalline structure, while volcanic rock is filled with pores of varying sizes. Even so, the accuracy of the calculation results is still acceptable. The fracture toughness of granite and volcanic rock is determined as 4.91 MPa·√m and 1.51 MPa·√m, respectively. It has been proven that the BEM and 4-p-b tests are effective in determining the fracture properties of quasi-brittle materials with various microstructures. 

### 4.3. Bamboo Scrimber

Bamboo scrimber is a type of unidirectional fiber composite derived from bamboo, prepared by compressing and bonding high-density bamboo strips. It shares a structure and properties akin to wood yet boasts greater hardness and compressive strength, thus serving as an environmentally sustainable alternative. Such material can be utilized for making structural components such as beams, columns, and flooring. Actually, the bamboo scrimber also belonged to the quasi-brittle material, and its fracture properties were tested with 3-p-b methods in the published literature [43]. The fracture test results are collected and summarized in Table 3 and Table 4. The specimen sizes and maximum failure loads are listed. It can be found that the bamboo scrimber specimens are set with different α-ratios and S/W ratios.

**Figure 9:**
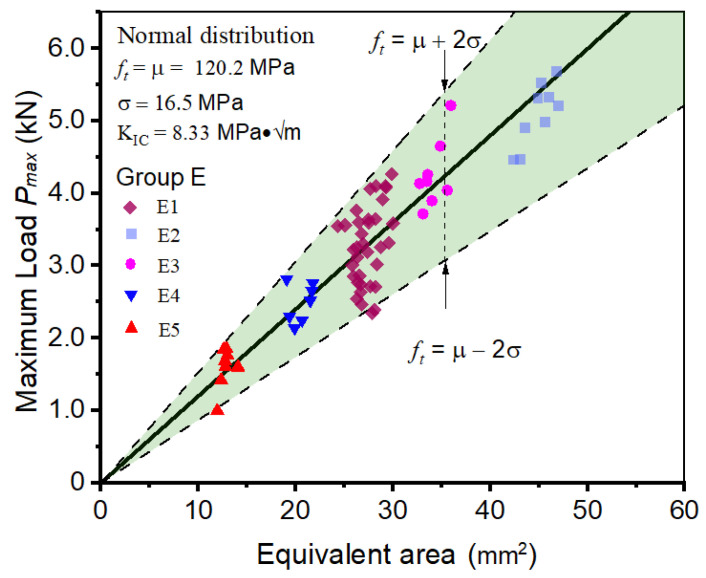
Determining the fracture properties of bamboo scrimber 3-p-b specimens in Group E.

The fracture properties of 67 specimens are calculated by inputting their fracture test results into the BEM model. Subsequently, an analysis of the distribution of tensile strength values revealed a mean value of 120 MPa and a standard deviation of 16.5 MPa; the standard deviation accounted for 13.7% of the mean value. By referring to Equation (22), the fracture toughness of bamboo scrimber is calculated as 8.33 MPa·√m. As illustrated in Figure 9, most data points are closely distributed around the solid line. This implies that the fracture properties acquired via the BEM and 3-p-b tests for bamboo scrimber are indicative and can effectively mirror the fracture characteristics of this material across various geometric conditions.

## 5. Discussion

As illustrated in Section 4, only three cases were listed in the current study, such as BEM and WS for concrete materials, BEM and 4-p-b for hard rocks, and BEM and 3-p-b for bamboo scrimber. Actually, the Boundary Effect Model has been applied in other scenarios [44,45,46], and a relatively accurate determination of material fracture properties has been witnessed. 

**Figure 10:**
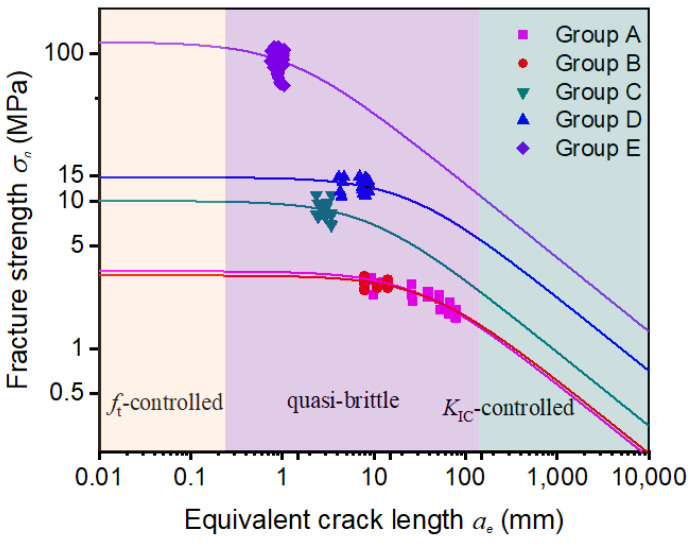
Using non-linear BEM to analyze the fracture failure types of concrete, hard rock, and bamboo scrimber specimens.

In this section, the non-linear BEM is used to analyze the above 140 fracture test results, including concrete, hard rocks, and bamboo scrimber. As shown in Figure 10, the fracture failure of materials could be divided into three regions: (1) tensile strength (*f_t_*) controlled region; (2) quasi-brittle fracture region controlled by both ft and *K_IC_*; and (3) fracture toughness (*K_IC_*) controlled region [37]. It could be found that all the test scatters concentrate in the quasi-brittle fracture region, despite the huge variation of material types, test methods, and specimen sizes. Hence, only the quasi-brittle fracture model, which considers the effect of microstructure, is suitable for determining the fracture properties of the above materials. 

**Figure 11:**
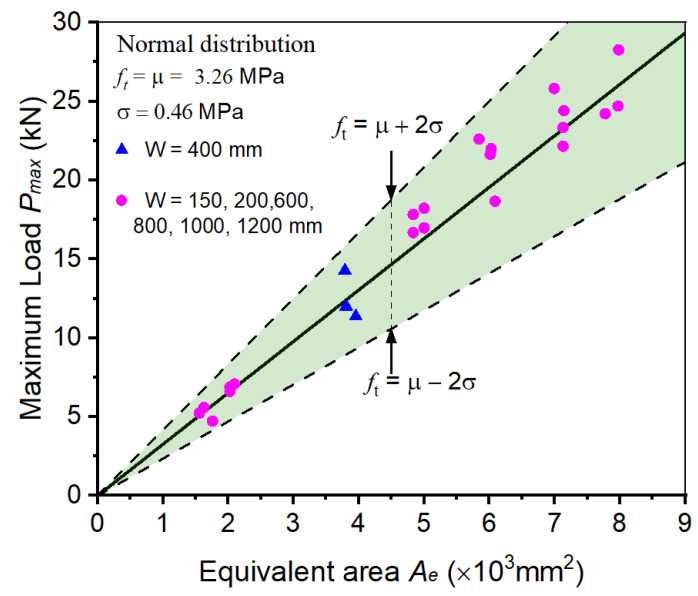
Applying linear-BEM to predict the fracture of concrete specimens with different sizes.

Then, the linear-BEM and three fracture test methods are adopted to predict the fracture load of quasi-brittle materials. As illustrated in Section 4, the fracture properties of quasi-brittle materials can be determined as constants regardless of the specimen sizes, specimen geometries, crack lengths, and material types. Moreover, the scattered distribution of fracture properties is utilized to establish the confidence interval of fracture prediction, which makes the fracture prediction more suitable for practical engineering. Taking the BEM and WS methods as an example, the test results of only three concrete specimens in Group A3 were used to construct the predictive model, and the remaining 35 specimens were utilized to verify the predictive performance of the model. As shown in Figure 11, most data in the other six groups fall in the predictive band; the fracture of concrete specimens in these groups is successfully predicted, regardless of the huge variation in specimen sizes. 

## 6. Conclusions

The fracture failure of quasi-brittle materials has attracted widespread attention from researchers, and various methods are proposed to analyze this phenomenon and calculate the fracture properties. In this study, the Boundary Effect Model (BEM) proposed by Hu is illustrated, and the concept, evolutionary process, and applicability scope are described in detail. After that, the BEM is combined with different fracture tests, and the calculating methods of fracture properties are applied to various quasi-brittle materials. The conclusions can be drawn as follows:

(1) The Boundary Effect Model attributes the well-known size effect to the influence of the specimen boundary and the microstructure of materials. Hence, to guarantee the accuracy and applicability of the fracture model, the fictitious crack around the notch tip, the distance between the notch tip and specimen boundaries, and the characteristics of material microstructure are all carefully considered during the construction of the Boundary Effect Model.
(2) On the basis of three-point bending (3-p-b) tests, four-point bending (4-p-b) tests, and wedge-splitting (WS) tests, the linear relationship between the maximum failure loads of the fracture test and material fracture properties is established.
(3) A total of 140 fracture test results for concrete, hard rocks, and bamboo scrimber were adopted to verify the reliability of the proposed methods. It is proven that the BEM-determined fracture properties can be considered material constants, regardless of the effect of specimen sizes, initial notch length, specimen geometries, microstructures, and material types.
(4) By virtue of non-linear BEM analysis, fracture failure is controlled by both the *f*_t_ and *K_IC_* criteria for most laboratory-conducted fracture tests of quasi-brittle materials. Combining the linear BEM and distribution characteristics of fracture properties, the fracture of quasi-brittle specimens can be accurately predicted.

## Figures and Tables

**Table 1:** The summarization of concrete fracture test results with the wedge-splitting method [40,41].

Group	*α*	*W*	*B*	*P_max_*	Group	*α*	*W*	*B*	*P_max_*
A1	0.46	150	180	4.7	A7	0.45	1198	200	24.7
	0.48	150	178	5.6		0.46	1200	201	24.2
	0.49	150	176	5.2		0.45	1200	200	28.3
A2	0.46	200	176	7.1	B1	0.353	170	200	10.7
	0.47	200	176	6.9					9.7
	0.47	200	176	6.6					11.0
A3	0.45	400	200	11.4	B2	0.471			6.6
	0.46	400	198	14.3					6.8
	0.46	400	199	12.0					7.0
A4	0.46	600	193	17.8	B3	0.588			4.1
	0.46	599	200	17.0					4.4
	0.46	600	193	16.7					4.9
	0.46	599	200	18.2					4.0
A5	0.45	799	196	18.7					4.5
	0.46	800	194	22.6					4.9
	0.46	798	200	21.6					5.0
	0.46	801	200	22.0					4.8
A6	0.45	997	200	22.1					
	0.45	997	200	23.3					
	0.45	999	196	25.8					
	0.45	1000	200	24.4					

**Table 2:** The summarization of hard rock fracture test results with the 4-p-b method.

Group	*α*	*W*	*B*	*P_max_*	Group	*α*	*W*	*B*	*P_max_*
C1	0.13	120	16	6.7	C4	0.67	120	16	1.3
	0.14			7.2		0.68			1.6
	0.15			8.2	D1	0.19	47	13	1.2
	0.15			6.3		0.21			1.1
	0.16			6.6		0.21			1.3
C2	0.26	120	16	5.2		0.23			1.7
	0.28			5.7		0.28			1.1
	0.29			5.7	D2	0.38	47	13	1.0
	0.31			5.4		0.40			0.9
	0.32			4.9		0.43			0.8
C3	0.42	120	16	3.6		0.43			0.8
	0.43			3.5		0.43			0.7
	0.43			3.4	D3	0.51	47	13	0.6
	0.46			3.5		0.53			0.5
	0.48			3.6		0.53			0.6
C4	0.64	120	16	1.9		0.55			0.5
	0.65			1.7		0.55			0.6
	0.66			1.3					

**Table 3:** The geometrical conditions of bamboo scrimber specimens.

Group	*α*	*W*	*B*	*S*	*S/W*
E1	0.1	15	20	60	4
E2	0.2	15	20	60	4
E3	0.3	15	20	60	4
E4	0.2	15	20	37.5	2.5
E5	0.2	15	20	120	8

**Table 4:** The summarization of bamboo scrimber fracture test results with the 3-p-b method.

Group	*α*	*P_max_*	Group	*α*	*P_max_*	Group	*α*	*P_max_*
E1	0.20	3.1	E1	0.18	3.7	E3	0.12	4.1
	0.18	2.4		0.15	3.6		0.11	3.7
	0.17	2.4		0.17	3.9		0.13	4.3
	0.19	3.2		0.20	2.9		0.10	5.2
	0.22	3.6		0.20	2.6		0.11	4.2
	0.18	4.1		0.20	2.8		0.10	3.9
	0.20	2.6		0.21	2.9	E4	0.29	2.5
	0.18	3.3		0.19	3.6		0.30	2.3
	0.17	2.7		0.21	3.2		0.33	2.8
	0.19	3.5		0.21	3.8		0.31	2.1
	0.18	3.6		0.18	4.1		0.28	2.7
	0.15	4.1		0.17	4.1		0.32	2.3
	0.18	2.7	E2	0.17	5.3		0.28	2.8
	0.16	4.3		0.18	5.0	E5	0.24	1.0
	0.17	3.3		0.17	5.2		0.22	1.9
	0.21	3.6		0.19	5.3		0.23	1.4
	0.19	2.5		0.18	5.5		0.21	1.7
	0.19	2.7		0.20	4.5		0.20	1.8
	0.17	3.0		0.17	5.7		0.18	1.6
	0.21	3.3		0.21	4.5		0.21	1.9
	0.21	3.0		0.19	4.9		0.21	1.6
	0.19	3.3	E3	0.09	4.7			
	0.22	3.6		0.09	4.0			

## Data Availability

Data are contained within the article.
